# Virus-Specific T Cells for the Immunocompromised Patient

**DOI:** 10.3389/fimmu.2017.01272

**Published:** 2017-10-11

**Authors:** Amy Houghtelin, Catherine M. Bollard

**Affiliations:** ^1^Program for Cell Enhancement and Technologies for Immunotherapy, Children’s National Health System, The George Washington University, Washington, DC, United States

**Keywords:** cell therapy, immunotherapy, adoptive, virus-specific T cells, immunocompromised host, *ex vivo* expansion, posttransplant complications

## Abstract

While progress has been made in the treatment of both hematologic cancers and solid tumors, chemorefractory or relapsed disease often portends a dismal prognosis, and salvage chemotherapy or radiation expose patients to intolerable toxicities and may not be effective. Hematopoietic stem cell transplant offers the promise of cure for many patients, and while mismatched, unrelated or haploidentical donors are increasingly available, the recipients are at higher risk of severe immunosuppression and immune dysregulation due to graft versus host disease. Viral infections remain a primary cause of severe morbidity and mortality in this patient population. Again, many therapeutic options for viral disease are toxic, may be ineffective or generate resistance, or fail to convey long-term protection. Adoptive cell therapy with virus-specific T cells (VSTs) is a targeted therapy that is efficacious and has minimal toxicity in immunocompromised patients with CMV and EBV infections in particular. Products have since been generated specific for multiple viral antigens (multi-VST), which are not only effective but also confer protection in 70–90% of recipients when used as prophylaxis. Notably, these products can be generated from either virus-naive or virus-experienced autologous or allogeneic sources, including partially matched HLA-matched third-party donors. Obstacles to effective VST treatment are donor availability and product generation time. Banking of third-party VST is an attractive way to overcome these constraints and provide products on an as-needed basis. Other developments include epitope discovery to broaden the number of viral antigens targets in a single product, the optimization of VST generation from naive donor sources, and the modification of VSTs to enhance persistence and efficacy *in vivo*.

## Introduction

While hematopoietic stem cell transplant (HSCT) offers a chance of cure for patients with many high risk cancers or primary immunodeficiency syndromes, transplant recipients remain vulnerable to infectious complications due to prolonged and profound immunosuppression (1–4). These risks are modified by preparative regimen, transplant type, and duration of myelosuppression (1–4). With advances in conditioning regimens and improved posttransplant management, an increasing number of patients are eligible to receive mismatched, unrelated, or haploidentical donor HSCT. While there have been great improvements in outcome for patients with severe or otherwise untreatable disease, the immunosuppression required for engraftment and, when indicated, to treat graft versus host disease (GVHD), opens the door for infection. In particular, viral infections cause significant morbidity and mortality, and the risk increases when T cell immune reconstitution is delayed (1–3). The relationship between immunosuppression, immune reconstitution, and the effects of GVHD, and infection are complicated and intertwined (5). Pharmacologic treatment and prophylactic options for viral infections remain limited and often ineffective, with associated morbidities notably from acute kidney injury and myelosuppression. Treatment may also generate resistance, and does not confer extended protection leaving patients at risk for viral reactivation (4). Given the correlation between delay in T cell immune recovery and viral disease, adoptive cell therapy is a logical alternative to pharmacologic therapy. Unmanipulated lymphocyte infusions from seropositive donors have been infused in patients with life-threatening disease such as EBV-associated lymphoma, demonstrating clinical efficacy with risks primarily associated with GVHD (6). This strategy has evolved over the past two decades, and donor lymphocyte products have been successful in reconstituting viral immunity in the host as a treatment for viral disease (including reactivation, new exposure, and lymphoma) and as prophylaxis (7). Following these initial studies, virus-specific T cell (VST) selection and/or expansion has been refined to maximize viral cytotoxicity and minimize alloreactivity to reduce and largely eliminate the risk of GVHD. In the current studies, VSTs offer targeted therapy and have demonstrated a very good safety profile to date (8–11). This review will detail developments in the manufacturing process, describe clinical success of VSTs and discuss future directions, including the use of naive donor sources and third-party banks.

## Materials and Methods

### Antigen Selection

To successfully generate and expand VSTs, specific immunogenic epitopes need to be defined for each pathogen. It is well established that some viruses, notably CMV and EBV, are known to have certain antigens expressed at various stages of disease (12–14). Using available tools, epitope mapping has allowed identification of immunogenic antigens for other viruses, including adenovirus, human herpes virus 6 (HHV6), and BK virus (15–18). For many of these viruses, the immunodominant and subdominant antigens have been characterized, as well as antigens which promote enhanced T cell proliferation and immune protection *in vivo* (19). Several methods have been used to expand and select VSTs. Most recently, antigen-presenting cells (APCs) exposed to peptide mixtures consisting of overlapping, 15mer peptide libraries have proved highly successful for direct stimulation of CD4^+^ and CD8^+^ T cells (17–19). Alternative approaches use APC exposed to whole virus, viral lysates, whole proteins, or viral vectors (7, 9, 15, 20–24).

### Antigen Presentation

Once an appropriate antigen has been identified as an immune target, it must be effectively presented by APC to T cells in conjunction with costimulatory signals to promote T cell activation and proliferation. The APC type impacts production time, cell numbers, and product phenotype. Examples include dendritic cells, monocytes, B cells, and various artificial APCs. Table 1 summarizes antigen and APC options. While dendritic cells are very effective APCs, they are limited by low numbers; thus, repeat stimulations require increasing amounts of donor cells. Whole virus has also been used to create potent APCs. For example, EBV lymphoblastoid cell lines (LCLs) infected with the B95-8 EBV strain, are effective and safe APC for generation of clinically useful products (9). However, this strategy is limited by lengthy incubation time with a potential risk of infection. Donor PHA blasts are potent, polyclonal stimulators of T cells and require only low cell numbers for generation (19). Artificial K-562 cells are another potential option for APC, and may be especially helpful to provide costimulation for generating VSTs from seronegative donors. This complementary costimulation in the presence of artificial APCs creates an effective antigen-presenting complex to promote VST stimulation and expansion (25).

**Table 1:** Antigen selection and presentation.

Antigen/APC	Advantage	Disadvantage
Whole virus/viral lysate	Potent antigen	Live virus, lengthy production time
Whole proteins	Readily available	Less potent antigen
Viral vectors	Reproducible	Lengthy production time
Peptide/peptide mixtures	Reproducible, standardized, readily available	Need identified immunodominant epitopes
Dendritic cells	Potent stimulators	Limited cell numbers, difficult to isolate
Monocytes	Easily isolated	Reduced potency
B cells	More robust numbers	Reduced generation of T_mem_ (26), increased production time
PHA blasts	Reduced production time, easily expanded	Moderate potency
Artificial APC	Easily expanded and maintained; effective costimulation	Varying efficacy

### T Cell Expansion

Initial attempts at adoptive immunotherapy used unmanipulated donor lymphocyte infusions to transfer cytotoxic and memory T cells specific for certain viral infections. While an effective antiviral strategy, a major complication was GVHD, a natural consequence from infusing alloreactive T cells (6, 27). Furthermore this strategy was essentially limited to EBV and CMV where high frequencies of VSTs circulate in the donor (28). Modifications in the generation and *ex vivo* expansion of these T cell products have minimized GVHD to an almost negligible risk (28, 29). These processes have evolved over time to select and expand VSTs while minimizing alloreactive or naive T cells in the final product, with the emphasis currently on reducing production times and maximizing product functionality.

#### Reduction of Alloreactive Cells

Several strategies have been aimed at inactivating or removing alloreactive T cells from donor products. One strategy evaluated blockade *via* monoclonal antibodies to the B7:CD28 costimulatory complex to produce an anergic response to recipient cells (30). This was successful, although time-consuming and not completely effective at preventing GVHD. Other strategies have employed selective depletion of alloreactive cells, either *ex vivo* or *in vivo*. *Ex vivo* methods use cells stimulated by recipient APCs followed by targeting alloreactive T cells through CD25, CD69, or CD95 and eliminating them by magnetic-coupled monoclonal antibodies or immununotoxins, photodynamic depletion, or apoptosis activation (31–34). These methods have seen some success *in vivo*, but results have been unreliable in terms of conveying viral protection and preventing GVHD. *In vivo* strategies employ a safety or suicide switch to deplete alloreactive T cells, to induce apoptosis in response to a specific signal. A thymidine kinase gene from herpes simplex virus I, acting as a trigger for cell elimination *via* ganciclovir exposure, was found to be effective but hampered by increased immunogenicity and a delay of several days to clinical effect after ganciclovir administration (35). Newer studies have transduced cells with the suicide gene-inducible caspase 9 (iC9), which triggers apoptosis after exposure to a dimerizing drug (36, 37). In this case, VSTs conveyed viral protection, and patients showed clinical improvement of GVHD symptoms soon after administration of the “safety switch” dimerizing drug.

#### Approaches for Selection and Expansion of VSTs

Isolation of VSTs with or without *ex vivo* expansion offers an alternative means of eliminating alloreactive cells. More recent methods have simplified this process to reduce production times and simplify manufacturing strategies.

##### Selection of VSTs

Virus-specific T cells may be isolated directly from donor peripheral blood with the use of peptide-HLA multimers to facilitate the identification and purification of antigen-specific T cells. This process was originally hindered by irreversible binding and significant changes in T cell phenotype. The use of streptamers greatly improved this method, acting as multimers that use an HLA-peptide complex to reversibly bind the desired T cells without altering T cell phenotype or functional status. While a major benefit of this method is the rapid availability of an antigen-specific T cell product, the selected T cells are limited by HLA-restriction imposed by the streptamer (38–40). This process also requires knowledge of defined Class I HLA-restricted viral epitopes for effective isolation, and it selects for a limited repertoire of CD8^+^ cells rather than the entire polyclonal, polyfunctional population of CD4^+^ and CD8^+^ T cells recognizing the full spectrum of available viral antigens. Despite noted limitations, investigators have been successful using VSTs isolated in this manner in the adoptive therapy for diseases with higher numbers of circulating VSTs, such as CMV and EBV. Such infused VSTs have also demonstrated expansion *in vivo* following transfer of these selected cells (41).

Interferon-γ (IFN-γ) capture also directly selects circulating VSTs from peripheral blood. Peripheral blood mononuclear cells (PBMCs) are stimulated with antigens specific for targeted virus and incubated over 4–16 h, inducing IFN-γ production in stimulated cells. A monoclonal antibody to IFN-γ coupled to a leukocyte-specific antibody (anti-CD45) then captures the IFN-γ producing cells, which are then selected *via* magnetic beads. This also allows rapid selection of VSTs free of HLA-restriction with the added benefit of stimulating and capturing a polyclonal population of CD4^+^ and CD8^+^ cells. This is clinically relevant, as the presence of CD4^+^ cells enhances the memory and effector response and supports persistence and expansion of the cytotoxic T cells (42, 43). It also allows for selection of VSTs responding to multiple viral epitopes and has been successful in generating clinically functional VSTs targeted to various viruses (44–47).

Both these capture methods allow for rapid and precise selection of circulating VSTs, with obvious benefits for timely treatment of patients with active disease. However, they require VSTs to be circulating at a detectable level and leukapheresis is often needed to collect clinically relevant cell numbers. These methods are thus not useful for naive donors and ineffective if numbers of circulating VSTs are too low to generate a useful product.

##### Expansion of VSTs

The process of *ex vivo* T cell selection and expansion has been refined over the past two decades, with an emphasis on decreasing production times and complexity and optimizing *in vivo* function. The first techniques using EBV-LCL lines required at least 3 months to generate the APC and make a product. Despite still taking at least 10 days, current VST culture expansion systems generate polyclonal and polyfunctional products, properties which enhance *in vivo* expansion, function, and persistence. Clinical trials using *ex vivo* stimulated and expanded VSTs show that infused T cells persist long-term, detectible by gene-marking studies for as long as 9 years (48, 49). *Ex vivo* stimulation and expansion requires only a small volume of blood to establish the culture, eliminating the need for costly, time-consuming, and invasive leukapheresis. Lastly, expansion cultures make possible the generation of VSTs from low levels of circulating VSTs and naive donor sources (19–21, 50). Expanded VSTs infused in post-HSCT recipients carries a potential risk of causing GVHD. While some studies have shown cross reactivity of these VSTs with recipient targets *in vitro*, no increase in either acute or chronic GVHD has been reported (51). In fact, even when mild cross-reactivity of expanded VSTs with HLA-mismatched targets has been demonstrated *in vitro*, it has not correlated with increased risk of GVHD *in vivo* (52). Further refinements in this process continue to evolve as these procedures become standardized, including the use of overlapping peptide pools and alternative APCs to improve reliability and reproducibility of products.

## Results of VSTs in Clinical Use

### CMV

Human cytomegalovirus is a pervasive β-herpes virus with prevalence rates of 50–100% in the general population. While it may manifest as mild self-limiting disease in the immunocompetent host, CMV can cause severe life-threatening disease in the immunocompromised host. Because CMV persists in the latent form after acute infection, CMV-specific CD4^+^ and CD8^+^ T cells are necessary to maintain viral quiescence. In post-HSCT patients, in the absence of donor immunity and in other immunodeficient states, CMV may reactivate in the form of retinitis, pneumonitis, hepatitis, or enterocolitis (53). The adoptive transfer of CMV-specific T cells is a logical strategy for treating and preventing CMV reactivation in such individuals, and numerous clinical trials confirm the overall excellent efficacy of VST (10, 41, 46, 54–60). CMV-specific VSTs generated from naive T cells in umbilical cord blood (UCB) have also proved effective. These VSTs show specificity for atypical epitopes while maintaining functionality (21). Naive donor sources such as UCB are being explored as a source for other VSTs for generating third-party banks for on-demand use as well (see Other Viruses and Third-Party VST Products) (21).

### EBV

EBV is a ubiquitous, highly immunogenic γ-herpesvirus that can cause unique complications following transplant. Over 90% of the general population have been infected and retain lifelong seropositivity. Manifestations of primary EBV infection vary widely from asymptomatic infection to a debilitating viral illness (61). Thereafter in most cases, EBV remains latent lifelong in a B cell and mucosal epithelial reservoir under continuous T cell immune surveillance. In these healthy individuals, up to 2% of circulating T cells are EBV specific. In the period of immune deficiency after HSCT, EBV reactivation may cause viremia and life-threatening posttransplant lymphoproliferative disease (PTLD). While the monoclonal antibody rituximab successfully treats severe EBV disease in many patients by eliminating B cells in which the EBV virus resides, it results in long-term reduction in antibody production and is not always successful at controlling PTLD (61). Adoptive T cell therapy for PTLD is facilitated by the high probability of finding healthy EBV-exposed donors with measurable frequencies of circulating EBV-specific T cells. First attempts using donor lymphocyte infusions to treat EBV-PTLD were complicated by high rates of GVHD (6). Subsequent *ex vivo* strategies to select and expand EBV-specific T cells show broad efficacy and safety of EBV VST in numerous clinical trials for prevention and treatment of viremia and PTLD (47–49, 62–68).

### Adenovirus

Adenovirus infection can range from mild upper respiratory tract infections to a spectrum of life-threatening pneumonia, gastrointestinal, hepatic, renal, and neurologic complications. Following infection, latency is maintained in the lymphoid tissues, but the virus can reactivate during periods of prolonged absence of T cell immunity (69). Adenovirus causes potentially lethal viral complication in post-HSCT recipients. Antiviral drugs such as ribavirin are largely ineffective. However, adenovirus-specific T cells generated from healthy donors have proven effective at treating even advanced disease (45, 70, 71). For this reason adenovirus antigens are often incorporated in the generation of multivirus-specific T cell products (see below).

### Other Viruses

The BK and JC polyomaviruses, normally latent in healthy tissues of most adult individuals, reactivate after HSCT and in immunodeficient individuals (72). BK virus may manifest as nephropathy and life-threatening hemorrhagic cystitis (HC). Rarely, the closely associated JC virus causes fatal brain damage from progressive multifocal leukoencephalopathy (73). Polyoma-specific VST are being developed to combat these viruses. A single case report describes the successful use of BK VSTs, after which the patient had complete resolution of HC without bystander organ toxicity, GVHD, or graft rejection (74). It is now clear that the platforms developed for *ex vivo* selected and expanded VSTs are readily adaptable to many other viruses that complicate immune deficient states, and future developments include developing VST to target an array of viruses including VZV, HHV, and even HIV (16, 75–79).

### VSTs Targeting Multiple Viruses

Given the success in prophylaxis and treatment of individual viral infections with single-virus-specific VST, the targeting of multiple viruses in a single product is a logical extension for managing the post-HSCT patient at risk from multiple viral infections. Several groups have successfully manufactured multivirus-specific T cells for the more common viruses (11, 76, 80). Challenges, as with single virus-specific products, include production time, labor, and cost. Several groups have increased manufacturing efficiency through use of viral plasmids, standardized pepmixes, alternative APCs, and alternative donor sources such as UCB to produce polyclonal, clinically efficacious VSTs (19, 81, 82). A potential obstacle for multivirus pepmixes is the risk that the most immunodominant antigens will outcompete other T cell expansions and dilute the final product of clonal diversity. Various ways to maintain multiviral specificity are being explored (80, 83). To broaden the applicability of multivirus VST, Hanley et al used virus naive donors and UCB sources to generate tri-virus-specific T cells with success (20, 84). The viral repertoire of multi VST products is continually being extended and there is no apparent limit to the number of viruses that could be targeted in a single product. As an example, Gerdemann et al established a Good Manufacturing Practices (GMP) grade method for generating VST targeting seven different viruses: CMV, EBV, adenovirus, BK virus, HHV6, RSV, influenza (19). More recently, the group at Children’s National has established a rapid, reproducible method in GMP-compliance for generating VSTs to CMV, EBV, Adenovirus, and BK virus from naive (cord blood) donor sources (82), paving the way for establishing third-party VST banks for “off the shelf” distribution.

### Third-Party VST Products

One of the more exciting developments in VST therapy is the generation of third-party VST banks. The development of a bank of efficacious, clinical-grade cell therapy products which pass all release testing requires an initial outlay in time, labor, and cost. However, immediate product availability avoids any risky delay in treatment of life threatening viral disease. Several groups have created third party VST banks for “off the shelf” administration. Since these products are derived from unmatched donors and not autologous or HLA-matched sources, they carry an increased risk of GVHD. Nevertheless, with attempts to match at least one HLA molecule with the recipient, third-party VST products have been successful in several clinical trials (Table 2) (85, 86). Predictably higher number of HLA matches between the VST product and the recipient (particularly HLA class I) correlate with better *in vivo* proliferation and superior efficacy (86). However, even less closely matched products can be effective despite limited persistence of these mismatched T cell products. In one study, third-party cells were identified up to 12 weeks after infusion and approximately 70% of VST recipients benefited (22). Reassuringly there is no indication of enhanced alloreactivity from the VST as measured by GVHD or graft rejection. Third-party banks are thus emerging as a promising option for treating refractory post-transplant viral infections.

**Table 2:** VSTs in clinical trials.

Target	*N*	Method of T cell selection	Antigen presentation	GVHD occurrences	CMV status	Reference/institution
CMV	18	IFN-γ capture	Peptide mixes of pp65	3 patients with grade I aGVHD; 3 patients with grade II/III aGVHD; 3 patients with cGVHD	11 developed CMV reactivation, all responded to antivirals or repeat infusion of T cells	(46)/UCL^a^

	7	*Ex vivo* expansion	CMV lysate and peptide mixes of pp65	No GVHD	Only 1 patient had persistent CMV viremia, one reactivation after steroids; CMV-specific T cell expansion in 6 patients	(54)/MKP^b^

	14	*Ex vivo* expansion	Dendritic cells with CMV-infected fibroblasts; only CD8 clonal population infused	3 patients developed grade I/II aGVHD, all responding to steroids	No CMV disease, CMV immunity restored	(55)/FHCRC^c^

	16	*Ex vivo* expansion	Dendritic cells with CMV-infected fibroblasts	3 patients with grade I aGVHD only	8 patients also required ganciclovir but subsequently cleared viremia; 2 patients developed CMV reactivation postinfusion; CMV immunity restored	(10)/UCL

	25	*Ex vivo* expansion	CMV antigen; only CD4 clonal population infused	1 case of GVHD	7 patients with CMV reactivation; 5 patients with clinical disease; 2 patient deaths from CMV	(56)/U of Perugia^d^

	18	IFN-γ capture	pp65 protein	1 case of GVHD	4 patients died of CMV-related disease; 15 patients with *in vivo* expansion	(57)/UCH^e^

	7	*Ex vivo* expansion	Dendritic cells with peptide mixes (pp65, IE1)	No GVHD	4 patients cleared CMV; 2 with reactivation (1 associated with high dose steroids), 1 with transient increase in CMV PCR	(58)/PSHCH^f^

	9	*Ex vivo* expansion	Dendritic cells with peptide mix (pp65)	3 patients with grade III aGVHD, with one associated death; 2 patients with cGVHD	2 patients with reactivation not requiring treatment	(59)/U of Sydney^g^

	16	*Ex vivo* expansion	Dendritic cells with peptide mix (pp65)	No GVHD	14 patients cleared CMV	(60)/MSKCC^h^

	2	Streptamer-selection	PBMCs with pp65-HLA beads	No GVHD	Both cleared CMV with CMV-specific expansion	(41)/U of Ulm^i^

EBV	39	*Ex vivo* expansion	PBMCs with LCLs	No aGVHD or new cases of GVHD	EBV-specific immunity restored, clearance of viremia, no PTLD	(49)/SJCRH^j^

	10	IFN-γ capture	EBNA1 overlapping peptide mixtures	1 patient with Grade I/II aGVHD	Expansion of EBV-specific T cells in 8 patients and clinical/virologic response in 7 patients	(47)/UCH

	6	IFN-γ capture	EBV peptide mix	No GVHD	Resolution of PTLD in 3 patients; progression of PTLD in 3 patients (all late stage at time of transfer)	(63)/HZM^k^

	114	*Ex vivo* expansion	PBMCs with LCLs	No *de novo* GVHD; 8 patients with reactivation of Grade I/II GVHD; 11 patients with limited cGVHD; 2 patients with extensive cGVHD	No PTLD development; remission of preexisting PTLD in 11 of 13 patients	(48)/BCM^l^

	19	*Ex vivo* expansion	T cells with LCLs	No GVHD	Resolution of PTLD in 13 patients; 2 patients with PD received DLI and 1 achieved CR	(64)/MSKCC

	36	*Ex vivo* expansion	T cells with LCLs	No aGVHD, 4 patients with limited cGVHD	No PTLD development	(65)/SJCRH

	42	*Ex vivo* expansion	T cells with LCLs	No GVHD	No PTLD development, reconstitution of EBV-specific immunity	(66)/SJCRH

	4	*Ex vivo* expansion	PBMCs with LCLs	No GVHD	Clearance of PTLD or EBV viremia	(67)/U of Pavia^m^

Adenovirus	9	IFN-γ capture	Adenovirus antigen C	Exacerbation of preexisting skin GVHD	5 patients responded with expansion of adenovirus-specific T cells in 5 patients	(70)/UCH

	30	IFN-γ capture	Hexon protein	2 grade I GVHD; overall decrease in patients with GVHD	21 patients responded	(45)/UCH

	1	IFN-γ capture	Hexon protein	No GVHD	Complete response	(71)/BGCH^n^

BK virus	1	IFN-γ capture	Large-T, VP1	No GVHD	Complete response	(74)/HH^o^

**Multivirus specific**						

EBV-CMV-Adeno	10	*Ex vivo* expansion	Dendritic cells nucleofected with viral plasmids: EBV (LMP1, LMP2, bzlf), CMV (IE1, pp65), adenovirus (hexon, penton)	1 grade I/II GVHD	8 patients with CR; 1 patient with stable EBV disease without PTLD	(81)/BCM

EBV-Adeno	12	*Ex vivo* expansion	PBMCs with Ad5f35 vector and LCLs	No GVHD	Expansion of virus-specific immunity, resolution or prevention of clinical disease	(11)/BCM

EBV-CMV-Adeno	11	*Ex vivo* expansion	PBMCs with LCLs transformed with Ad5f35-CMVpp65 vector	No GVHD	Expansion of EBV- and CMV-specific immunity in all patients, adenovirus-specific immunity in patients with clinical disease; clearance of all clinical disease	(80)/BCM

EBV-CMV-Adeno-VZV	10	*Ex vivo* expansion	PBMCs with Ad5F35-pp65, Ad5F35-EBNA1/LMP, VZV vaccine	1 grade II GVHD, 1 grade III GVHD	6 patients with CMV reactivation, only one receiving antiviral therapy; no EBV, adenovirus, or VZV reactivation	(76)/U of Sydney

EBV-CMV-Adeno-BKV-HHV6	11	*Ex vivo* expansion	PBMCs with pepmixes (LMP2, BZLF, EBNA1, penton, hexon, pp65, IE-1, VP1, large T, U11, U14, U90)	1 grade II aGVHD	No viral reactivation in 3 patients infused prophylactically; EBV—5 patients with CR, including PTLD; CMV—2 patients with CR, 1 PR; adenovirus—1CR; BKV—5 patients with CR, 1 PR, 1 NR; HHV6—2 patients with CR	(75)/BCM

**Third party**						

EBV	8	*Ex vivo* expansion	PBMCs with LCLs	No GVHD	3 patients with CR; 1 patient with PR, subsequently refused treatment; 2 patients with no response; 2 patients passed away before evaluation (unrelated to VSTs)	(85)/U of Edinburgh^p^

EBV	33	*Ex vivo* expansion	PBMCs with LCLs	No GVHD	21 patients with CR or PR; 6 month OS 79%	(86)/U of Edinburgh

EBV-CMV-Adeno	50	*Ex vivo* expansion	PBMCs with LCLs transformed with Ad5f35-CMVpp65 vector	6 with grade I GVHD; 1 with grade II GVHD, 1 with grade III GVHD	17 of 23 with PR/CR for CMV; 14 of 18 PR/CR for adenovirus; 6 of 9 PR/CR for EBV	(22)/BCM

EBV	2	*Ex vivo* expansion	PBMCs with LCLs	No GVHD	Both with CR	(87)/MSKCC

*N = number of patients in study*.

*^a^University College London, London, England*.

*^b^Medizinische Klinik und Poliklinik, Tübingen, Germany*.

*^c^Fred Hutchinson Cancer Research Center, Seattle, WA, USA*.

*^d^University of Perugia, Perugia, Italy*.

*^e^University Children’s Hospital, Tübingen, Germany*.

*^f^Penn State Hershey Children’s Hospital, Hershey, PA, USA*.

*^g^University of Sydney, Sydney, NSW, Australia*.

*^h^Memorial Sloan-Kettering Cancer Center, New York, NY, USA*.

*^i^University of Ulm, Ulm, Germany*.

*^j^St. Jude Children’s Research Hospital, Memphis, TN, USA*.

*^k^Helmholtz Zentrum München and Ludwig-Maximilians-Universität, Munich, Germany*.

*^l^Baylor College of Medicine, Houston, TX, USA*.

*^m^University of Pavia, Pavia, Italy*.

*^n^Bambino Gesù Children’s Hospital, Rome, Italy*.

*^o^Hammersmith Hospital, London, UK*.

*^p^University of Edinburgh, Edinburgh, UK*.

## Discussion and Future Directions

Over the past two decades, VST treatment has evolved from first proof of principle to a broadening acceptance that these cell products are a valuable, low-risk and effective tool to treat viral infections in immunocompromised individuals. Response rates reach approximately 90% for patients post-HSCT receiving VST from the matched transplant donor, and are around 70% for patients receiving third-party VST products (Table 2). VSTs appear promising as prophylaxis for high risk patients, conferring a high probability of protection against reactivation. A recent review of VSTs given to 36 patients with primary immunodeficiency syndromes over the past 10 years reported excellent responses to VSTs both for treatment (response rates 76–100% depending on the virus) and prophylaxis (81% of patients protected from viral reactivation) (24). Improved technology, including standardized pepmixes and alternative APCs, has improved the speed and efficiency of the manufacturing process. In parallel, the successes with VST from naive donor sources, multivirus-specific products, and generation of third-party banks have widened the scope of VST applicability. Ongoing studies are evaluating the safety and feasibility of increasing the number of viruses in a single product and extending the size of third-party banks for rapid use. Given the success seen in viral infections, the expansion of antigen-specific adoptive cell therapy to other complicated diseases, including HIV, fungal disease, and malignancies is increasingly within reach.

The studies included in Table 2 demonstrate the safety of VSTs in various settings. The risk of GVHD, a primary concern in initial trials using unmanipulated donor products, has been decreased by improved methods of selecting and expanding VSTs. Current GVHD rates after VST do not exceed those expected for patients post-HSCT. Of studies with particularly high rates of GVHD, nearly all of the patients who developed GVHD (both acute and chronic) had prior risk factors that would explain these outcomes, including history of or active GVHD, subtherapeutic immunosuppression, or recipients of T cell-replete grafts (46, 59). Critical, taking into consideration the patient-specific risk factors, no correlation has been identified between GVHD development and the method of VST generation, product phenotype, or duration of *in vivo* activity of infused VSTs.

Through the multitude of clinical trials utilizing VSTs, we have gained some important information regarding predictors of response. Most methods of generating antigen-specific T cells yield a very heterogenous population of CD4^+^ (typically about 30%) and CD8^+^ T cells, unless they are generated against a single CD8^+^-restricted epitope. While we know this polyclonal phenotype supports persistence of VSTs in vivo (43), it is not clear whether differing proportions of CD4^+^/CD8^+^ T cells are associated with increased or decreased clinical efficacy. One special situation is the use of third-party products, where it appears critical to ensure that there is shared antiviral activity through a shared HLA allele when selecting the “right” product. This can be either class I or class II for most cases, although the endogenous immune response is HLA-specific for certain viruses and must be matched accordingly. For example, the response to adenovirus is mediated through HLA class II, thus products for patients with adenoviral disease should be matched through HLA class II, whereas for CMV class I matching is typically preferred.

Immune reconstitution studies in patients following infusion of VSTs have also lead to increased knowledge about the *in vivo* activity of different VST products. For latent viral infections (e.g., CMV and EBV), enhanced detection of circulating antiviral T cells has been correlated with a better response (10); however, this is not always the case for viruses that are not latent (e.g., adenovirus) (80). While the gene marking studies by Heslop and Rooney suggest that adoptively transferred EBV-specific T cells can persist for a decade, there is also the suggestion that adoptive transfer of VSTs can stimulate endogenous anti-viral immunity. Additionally, epitope spreading is another marker of improved immune response as illustrated in the EBV-associated lymphoma setting (88).

For all the successes observed with VST products over the years, some patients still fail to respond to therapy with no identifiable mechanism. Hence, an important area of ongoing research is evaluating the mechanisms underlying VST resistance. For example, tumor (or virus)-secreted TGFβ inactivates antigen-specific T cells. To overcome this obstacle, gene manipulation of the TGFβ receptor on the antigen-specific T cells to render them resistant to the effects of TGFβ is being explored (89, 90). Other groups have found success infusing galunisertib, a small molecule inhibitor of TGFβ, abrogating the anti-inflammatory effect (91). Targets may also evade the immune system by upregulating expression of immunomodulators such as programmed death-1 ligand, which binds PD-1 on T cells in response to IFNγ. Checkpoint inhibitors, such as pembrolizumab targeted to PD-1, have increasing applications to many malignancies or in combination therapies and may also enhance VSTs especially in the HIV setting (92). Such modifications may therefore increase the *in vivo* efficacy of adoptively transferred VSTs in patients post-HSCT or for virus-associated diseases.

Other challenges of treatment are related to the severe immune dysregulation in the majority of patients at risk of viral disease. Steroid treatment, often used in high doses to treat GVHD, can reactivate dormant viruses and also render VST infusion futile through inactivation of the product. To this end, VST have been gene manipulated to inactivate the glucocorticoid receptor, allowing them to maintain cytotoxicity in the presence of steroids (93). T cells can also be genetically manipulated to be resistant to calcineurin inhibitors (cyclosporine A and tacrolimus, commonly used in post-HSCT setting), which inhibit T cell activation *in vivo* (94, 95). Such transduced VSTs proliferate, lack alloreactivity, and maintain cytotoxicity.

Planned advanced phase clinical trials will focus on many of these crucial points, including delineating the importance of VST product phenotype for efficacy *in vivo*, overcoming challenges such as unfavorable microenvironment, and studying immune reconstitution markers following infusion. The application of adoptive cell therapy has become increasingly broad, now extending to other infections as well as hematologic malignancies and solid tumors. As we gain more information about the interplay between the host immune system and the disease, modifications to T cells or combination therapy approach may be increasingly used to maximize efficacy while maintaining safety. Current results and these future developments ensure that VSTs will have an increasingly successful and everwidening role in the management of the immunocompromised patient.

## Author Contributions

All authors have made a substantial, direct, and intellectual contribution to the work and approved it for publication.

## Conflict of Interest Statement

CB has a licensing agreement with Cell Medica. The other author declares that the research was conducted in the absence of any commercial or financial relationships that could be construed as a potential conflict of interest.
